# Measuring Attributions 50 Years on: From within-Country Poverty to Global Inequality

**DOI:** 10.3390/bs14030186

**Published:** 2024-02-26

**Authors:** Franco Bastias, Nadja Peter, Aristobulo Goldstein, Santiago Sánchez-Montañez, Anette Rohmann, Helen Landmann

**Affiliations:** 1 Cluster of Excellence “The Politics of Inequality”, University of Konstanz, 78464 Konstanz, Germany; franco.bastias@uni-konstanz.de; 2 National Scientific and Technical Research Council, Buenos Aires C1425FQB, Argentina; 3 Faculty of Psychology, FernUniversität in Hagen, 58097 Hagen, Germany; nadja.peter@fernuni-hagen.de (N.P.); anette.rohmann@fernuni-hagen.de (A.R.); 4 Faculty of Philosophy and Humanities, Catholic University of Cuyo, San Juan 5400, Argentina; aris.goldstein@uccuyo.edu.ar (A.G.); santiago.sanchez@uccuyo.edu.ar (S.S.-M.); 5 Department of Psychology, Universität Klagenfurt, 9020 Klagenfurt, Austria

**Keywords:** poverty, poor, attributions, measurement, inequality, global, systematic review

## Abstract

Fifty years after Feagin’s pioneering 1972 study, we present a systematic review of the measurement of attributions for poverty and economic inequality. We conducted a search for articles published from 1972 to 2023 in APA PsycArticles, Psychology and Behavioral Sciences Collection, APA PsycInfo, PSYNDEX Literature with PSYNDEX Tests, and Google Scholar. We used the following English keywords: “poor”, “poverty”, “inequality”, “attribution”, and “attributions” and their equivalents in Spanish. Applying our inclusion and exclusion criteria led to a final sample of 74 articles. We report three main findings. First, the majority of studies classify attributions on the dimensions of individualistic vs. structural. Second, there is a clear tendency to measure attributions for domestic poverty without considering supranational factors or poverty as a global challenge. Third, studies focus almost exclusively on poverty rather than (economic) inequality. We identify potential for future development within the literature, namely, from a domestic to a global perspective, from locus to controllability, and from poverty to inequality.

## 1. Introduction

According to the most recent human development report by the United Nations Development Programme, the number of people living in multidimensional poverty—that is, facing deprivations in health, educational opportunities and material standards of living—is as high as 1.3 billion today [1]. While the Human Development Index, measuring life expectancy, mean years of schooling, and gross national income per capita, dropped in most countries in the period from 2020 to 2021, countries with a lower index were more severely affected, highlighting the exacerbation of global inequalities due to recent crises such as climate change and the COVID-19 pandemic [2].

Psychological factors are relevant for the perpetuation of poverty and inequality since they influence (economic) behaviour and (political) preferences. Over the past decade, scholars have started examining the perceptions of global inequality, and in particular justice appraisals of global inequality [3,4]. Perceptions of global injustice have been found to be associated with behavioural intentions or actions directed at more global equality such as fair-trade consumption and environmental protection [5,6]. But when do people actually consider poverty and inequality as an injustice? One important factor influencing (in)justice perceptions is causal attribution.

In line with this reasoning, a wide array of research on poverty attributions demonstrates that the extent to which people support redistribution policies and welfare, engage in egalitarian action, or have intentions to help the poor very much depends on their causal explanations for poverty. In particular, those who perceive the causes of poverty to be rather structural, external to and uncontrollable by the poor embrace more egalitarian attitudes, as opposed to those who perceive the causes of poverty to be individualistic, internal to and controllable by the poor [7,8,9].

### 1.1. Measuring Attributions—A Brief History

One of the pioneering authors researching poverty attributions was Feagin [10], who classified explanations for poverty into three basic dimensions: (1) *individualistic*, including causes referring to dispositional and personal characteristics such as lack of will, motivation, or laziness; (2) *structural*, relating to environmental and societal factors like the job market, lack of opportunities, or discrimination; (3) *fatalistic*, covering explanations such as destiny, supernatural powers, and luck. During the 1970s, studies from India [11] and Australia [12] provided empirical evidence supporting Feagin’s claims and laying the foundations of the first taxonomy.

**Figure 1:**
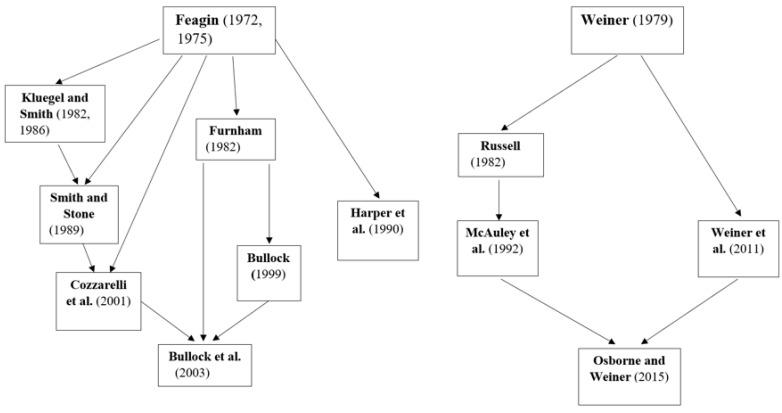
Theoretical influences of attribution measures [10,13,23,24,25,26,29,30,31,32,34,35,36,37].

Over the next years, Weiner and collaborators further developed and extended attribution theory [13]. They studied how people’s causal explanations affect their behaviour and emotions and proposed a different taxonomy for causal attributions with the following dimensions: (a) *locus,* referring to the internal or external qualities of a cause; (b) *stability* , indicating whether causes are stable or unstable over time; (c) and *controllability*, related to a person’s experienced control or agency over the situation and ranging from controllable to uncontrollable [14,15]. However, while this approach has been influential in research on causal explanations for achievement in educational settings [for reviews, see [16,17]] and for the plights of different stigmatised groups [18,19,20,21], to date, it has received only little attention in the literature on attributions for economic outcomes [15,22,23]. These different paths of theoretical influences are outlined in Figure 1. (Following Weiner’s description of the dimensions of locus, stability, and controllability [13], Russell created a semantic differential scale called *Causal Dimension Scale (CDS)* [24]. A decade later, McAuley and colleagues presented a revised version of the scale, the *Causal Dimension Scale II (CDSII)* [25]. More than twenty years later, a revised version of the scale was used to study causal attributions of poverty by Osborne and Weiner [22]).

At the beginning of the millennium, Cozzarelli and colleagues validated a 22-item scale that included internal, external, and what they introduced as *cultural attributions* [26]. The authors questioned the relevance of fatalistic attributions (i.e., beliefs that outcomes are determined in advance and cannot be changed, e.g., fate). To account for this critique, they suggested a dimension referring to a *culture of poverty* with items such as “born in poverty” or “dysfunctional nuclear family”. This new proposal for measuring attributions was widely applied in many subsequent studies [27,28]. A couple of years later, Bullock and colleagues developed a scale called the *Attributions for Poverty Questionnaire* [29], a revised and improved version of her previous scale [30]. This new scale includes some items from Cozzarelli and colleagues and Furnham [26,31] and contains 45 items designed to assess individualistic, structural, and fatalistic attributions. Additionally, items referring to further structural causes were incorporated, e.g., the gap between men and women, the lack of adequate childcare, and (lack of) parental support.

While most research in the realm of poverty attributions so far has focused on domestic poverty, in the early 1990s, some scholars developed an interest in the global dynamics of poverty. Harper and colleagues designed the *Causes of Third-World Poverty Scale* [32], which was later on translated and validated in other languages [33]. Their principal component analysis indicated the following four factors: blame the poor, blame third-world governments, blame nature, and blame exploitation. The last two factors are unique compared to the studies by Feagin [10,34]. The dimension “blame nature” includes items such as “their land is not suitable for agriculture”, and “blame exploitation” includes items such as “the world economy and the banking system are against the poor”.

### 1.2. The Present Research

Over the past decades, different instruments with a variety of dimensions have been developed to measure poverty and inequality attributions. However, the study of attributions for poverty has been criticised for several reasons [38,39,40]. Firstly, most measures focus on domestic poverty and are based on data from countries with relatively low poverty rates [41,42,43,44]. Hence, they are developed from what has been called a WEIRD perspective (Western, Educated, Industrialised, Rich, Democratic [45]) and do not consider poverty as a global issue. Secondly, as Weiner [40] (p. 604) points out, “attribution theory has been built upon the idea that causal beliefs reside within (internal to) or outside (external to) the person”, even though some objects of inquiry may “fall[s] in the middle of a locus dimension anchored with internal and external”. That might apply to poverty and inequality, where it is difficult to locate the cause inside or outside a person if we conceptualise the phenomena as a relationship between the individual and their context. Relatedly, current studies emphasise poverty rather than the concept of inequality. More specifically, examining inequality as opposed to poverty considers the advantaged as well as the disadvantaged group or person—an important aspect given the historical and societal mechanisms through which social injustice is perpetuated. Finally, some authors have discussed the fact that the poverty attributions scales are based on items developed several decades ago [33].

To date, however, the criticism regarding research on poverty attributions has rarely been based on a systematic review of the literature [46,47]. In the present study, we intend to close this gap by conducting a systematic review of the different scales used to measure attributions for poverty and economic inequality between 1972 and 2023. In particular, we examine the following research questions:

1. On which domains do the scales focus (i.e., attributions of domestic poverty, global poverty, domestic inequality, or global inequality)?
2. On which theoretical approaches are the scales assessing poverty and inequality attributions based?
3. Which dimensions of attributions are covered?
4. In what countries were the samples collected?

## 2. Materials and Methods

A search was carried out for articles published in the period between 1972 and 2023 using the databases APA PsycArticles, Psychology and Behavioral Sciences Collection, APA PsycInfo, PSYNDEX Literature with PSYNDEX Tests, and Google Scholar. The final search was carried out in December 2023 (see Table A1, in the Appendix A). The keywords used were *poor*, *poverty*, *inequality*, *attributions* and *attribution* and their equivalents in Spanish: *pobre*, *pobreza, desigualdad*, *atribuciones* and *atribución*. These words were combined as follows: *poor + attributions; poverty + attributions; inequality* + *attributions; poor + attribution; poverty + attribution; inequality* + *attribution*. A filter was used such that the keywords had to appear in the title of the article.

**Figure 2:**
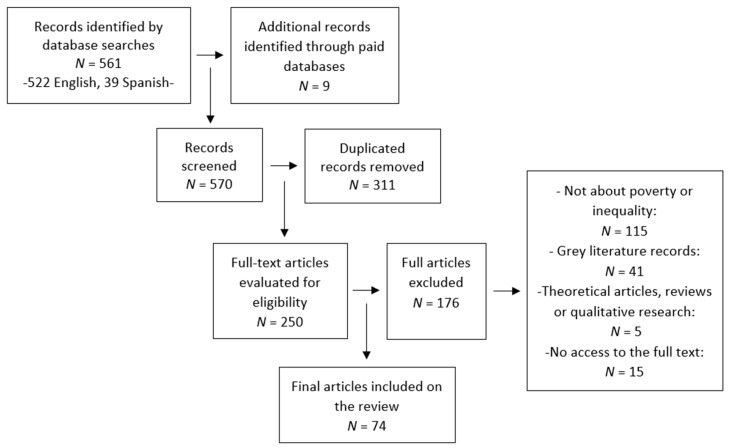
PRISMA flow diagram of the systematic literature search.

During the selection process (see Figure 2), some articles were discarded in line with the following exclusion criteria: unpublished undergraduate and graduate theses, conference papers, qualitative studies and papers that did not use scales to measure the construct. For the search referring to inequality, we also excluded any articles referring to gender inequality and other types of inequality beyond economic, wealth, and income inequality. Finally, a list of 74 articles was obtained, of which 63 were written in English and 11 in Spanish.

## 3. Results

We analysed articles concerning the following information: author and year of publication, country of origin of the samples, scope of attribution, sample size and properties, theoretical reference for the scale employed, number of items in the scale and scale response anchors, scale dimensions and their respective reliability. Appendix A details the information collected.

### 3.1. Sample Characteristics

As shown in Appendix A, most of the identified articles were written in English, while 14.9% were written in Spanish. Regarding the continent of origin of the samples included, 27 studies used samples from North America (with only 2 studies from Mexico), 23 from Europe, 14 from Asia, including Turkey and the Middle East, 7 from South America, 5 from Oceania, 5 from Africa, and 2 from Central America. Hence, approximately 65% of the research collected data in Europe, Canada, or the US. A total of 29 studies surveyed university students. All articles except 12 used Likert-type scales to measure attributions (see Appendix A).

### 3.2. Theoretical Influences

The attributional processes involved in understanding poverty and inequality can be considered a phenomenon that is psychological, sociological, and political in nature. Consequently, over the last 50 years, authors from the fields of psychology (e.g., Adrian Furnham, Bernard Weiner, Catherine Cozzarelli, David J. Harper, Heather Bullock), sociology (e.g., James R. Kluegel, Joe Feagin), and political science (e.g., Kevin B. Smith, Wim van Oorschot) have engaged in dialogue with each other through their work to understand how people explain poverty and inequality. Thus, in our review, despite focusing mainly on psychological studies of attribution processes, the underlying dialogue with other disciplines was revealed in different ways. For instance, a few articles from other social science disciplines besides psychology emerged in the search [48,49], and we were able to identify influences from other disciplines in psychology studies, mainly political science [50,51] and sociology [10,35,36].

**Figure 3:**
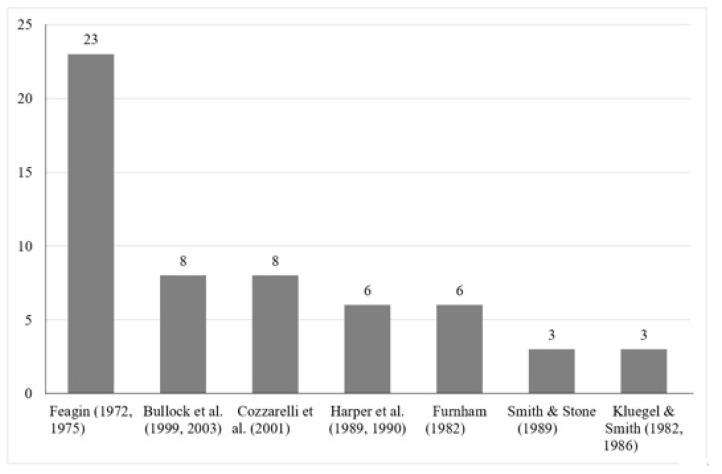
Occurrence of different theoretical perspectives among the reviewed articles [10,26,29,30,31,32,34,35,36,37,38].

As depicted in Figure 3, a large majority of the studies were explicitly guided by Feagin’s theoretical proposition and employed his scale either in its original version [10] or retained an indirect influence by employing scales designed based on Feagin’s work, such as those by Cozzarelli et al. [26], Bullock et al. [29,30], and others (see Figure 1).

Only six studies took a more global perspective by employing the *Causes of Third-World Poverty Questionnaire* developed by Harper and colleagues [32], who also developed their items based on Feagin [10]. Eight studies employed the *Attributions for Poverty Scale* by Cozzarelli et al. [26], which was based on Smith and Stone [37]. This scale places particular emphasis on the cultural aspect of poverty. Another eight studies opted for the *Attributions for Poverty Questionnaire* [29,30], which also drew inspiration from Cozzarelli et al., and Furnham [26,31]. Furnham’s measure was employed six times by different authors [31]. Three works used the *Attributions for Poverty Questionnaire* by Kluegel and Smith [35,36]; they employed a version of Furnham’s questionnaire [31] in addition to Feagin’s scale [10]. Furthermore, another three studies employed the *Beliefs About Poverty Scale* designed by Smith and Stone [37] based on both Feagin [34] and Kluegel and Smith [35,36]. Finally, two studies opted for the scale designed by van Oorschot and Halman [50], who drew inspiration from Kluegel and Smith [35,36].

### 3.3. Dimensionality of Poverty and Inequality Attributions

Since the vast majority of studies and scales descended directly or indirectly from the work of Feagin [10,34], the dimensionality of the scales used follows the original three-category typology of poverty explanations. As shown in Appendix A, 40 studies employed individualistic and structural dimensions. (Note that among the studies reviewed, individualistic is sometimes called individual, individualism, and dispositional; while structural is at times renamed as structuralistic, sociostructural, societal, or situational. Despite the varying terminology, the original meaning of the two dimensions by Feagin [10] is largely preserved.) The fatalistic dimension was present in 30 articles as well. By contrast, the distinction between internal and external factors, mainly following Weiner’s approach [15,22,23], was employed in 22 studies. Furthermore, factor structures based on Harper [32,38] were found in four studies, with dimensions such as blame exploitation, blame the poor, blame conflict, blame nature, and blame the governments of third-world countries (see Appendix A). Finally, the cultural dimension was present in nine papers. Only a few studies contained other dimensions, such as guilt/fate [50]; motivation [51,52]; family and morality [53]; problems in romantic relationships and related to having children [54]; chance [55]; competition, social attractiveness, and physical attractiveness [56].

## 4. Discussion

Our systematic review provides an overview of measurements of attributions for poverty and economic inequality employed in psychological studies. It highlights the focus on domestic poverty compared to global poverty or global inequality as well as the dominance of specific theoretical approaches and WEIRD samples. We identify a potential for further development within the literature, namely, from a domestic to a global perspective, from locus to controllability, and from poverty to inequality, which are discussed in the following sections.

### 4.1. From A Domestic to A Global Perspective

Most of the studies that resulted from our search focused on domestic or within-country poverty. In fact, several of the cross-national studies found are concerned with comparing how the populations of different countries explain internal economic problems within their own borders [57,58], rather than examining how global poverty and inequality between countries are explained. Of the few exceptions we identified as taking a global perspective, all were based on the *Causes of Third-World Poverty Scale* developed by Harper et al. [32], who in turn had based their items on Feagin [10]. Among those, Hine and Montiel are the only ones to have added a few items to the scale based on actual qualitative data, namely interviews with NGOs in Canada and the Philippines [7]. Moreover, except Vázquez and colleagues [59], all articles were published more than ten years ago. Thus, it can be assumed that the respective scales do not accurately reflect the current political and societal context. This becomes even more clear when looking at the actual items of the *Causes of Third-World Poverty Questionnaire* [7,32] in more detail, some of which use language that can be considered offensive from today’s perspective. The same is true for the very name of the scale, referring to the “third world”, which is not considered appropriate any longer. These results point to the need for an up-to-date scale.

The lack of a global perspective in studies on poverty and inequality attributions is evident in two additional aspects. First, supranational factors are scarce to non-existent among the most common causes suggested by researchers and listed in their scales for respondents to explain their domestic economic issues (for the list of causes, see [23]). As Baute & Pellegata [60] show in their study, in multi-level governance systems, citizens can attribute responsibility for economic results to supranational institutions, such as the European Union. Second, we found that WEIRD samples are overrepresented in inequality and poverty attribution studies, at least among top journals. About 65% of the research reviewed was based on data from Europe, Canada, or the US. There was a particularly strong bias in favour of US samples, at 23 out of the 74 articles resulting from our search. In contrast, Asia, Latin America, Oceania, and Africa were represented with only a small number of studies each.

This is in line with previous criticism of psychological research overall and research on poverty attributions in particular. With regard to the former, analyses of publications in six top psychological journals from 2003 to 2007 by Arnett [61] and from 2014 to 2018 by Thalmayer and colleagues [62] have shown that even though the percentage of studies based on US samples has decreased over the past decade or so, this is mainly due to an increasing number of studies using samples from other English-speaking countries and Western Europe. The majority of the world, on the other hand, is still severely underrepresented. Only 4–5% of the studies in the journals analysed were based on samples from non-WEIRD countries, even though they represent 89% of the world’s population [62].

These numbers are especially concerning with regard to the topic of poverty and inequality attributions. The parts of the world which, in global comparison, are most affected by the issues of poverty and most disadvantaged by global inequality are the ones least represented in research on this topic. This has previously been discussed concerning Asian countries [41,43] and Latin America [42,44] and holds equally true for the African continent. Overall, global poverty and inequality are urgent matters that deserve to receive more attention from attribution researchers. Our results highlight the need for an up-to-date scale grounded in the current societal context and taking into account perspectives from different parts of the world. Future research should further investigate whether the dimensions of poverty and inequality attributions replicate across different countries and cultures with scales adapted for the respective context.

### 4.2. From Locus to Controllability

Our findings show that 50 years after Feagin’s pioneering study on poverty attributions [10], his approach still remains very influential in the field. The vast majority of the reviewed studies are based on his tripartite operationalisation of attributions with the individualistic, structural, and fatalistic dimensions. However, we point out that this original three-part typology has its shortcomings. On the one hand, the fatalistic dimension does not seem to be as relevant as the individualistic and structural ones [35,36,63,64,65,66,67] and has been criticised for its low reliability [8,29,67,68,69,70,71]. (In particular, when “the poor” are referred to in more specific categories or characterised in certain ways (e.g., immigrants, families with children, the elderly), the distinction between individualistic and structural explanations remains for the different groups, while it is rare for the fatalistic dimension to emerge [39].) On the other hand, the individualistic dimension conflates causes that could be the individual’s fault and others that are not, despite this being a fundamental nuance to make when it comes to understanding people’s reactions to poverty [23,72]. Why do physical disability, laziness, and drug use, all internal causes of poverty, give rise to very different reactions [72]? We develop this point below.

Many authors stick to Heider’s [73] original proposal calling the individualistic–structural pair internal–external [48,74] and employing them practically as synonyms [75]. Nevertheless, following Weiner [22,23], we believe that talking about internal–external represents a new configuration of causal beliefs, more complex than Feagin’s proposal, since it implies defining internal or external to what. That is, if the cause of poverty is internal or external to the person, one is referring to locus; if the cause of poverty is internal or external to the person’s agency, one is referring to controllability. (In his 2 × 2 × 2 taxonomy, in addition to locus and controllability, Weiner proposes a third dimension: the stability of the causes. The stability of poverty over time and generations may suggest that the issue goes beyond the control of the individual, as Osborne and Weiner showed by reporting a negative correlation between stability and personal control [22]. However, this study also indicated the scarce relevance of this dimension as a predictor of personal helping behaviour.) Beyond individualistic–structural differentiation, this classification of locus and control effectively opens new possibilities to explore how people (including researchers) understand poverty as a highly complex phenomenon, where it is common to erroneously consider correlates as causes of poverty and where in certain conceptual definitions of the phenomenon, such as multidimensional poverty [76,77], the correlates may be better described as dimensions of poverty.

In particular, Weiner’s taxonomy allows us to explore explanations of poverty that conceive that the causes are located within the person, but are external in their controllability, such as lack of education, child malnutrition, disabilities, etc. This differentiation is especially important for capturing intergenerational explanations of poverty. My parents’ deprivation might result in my poor access to education, leading to my impoverishment. My own poverty might then be attributed internally to my lack of education, while in fact the cause—my parents’ poverty—is external and controllability lies within external factors like the school system. As in this example, the causes of poverty are often located within the poor when they are confused with the traces poverty leaves on the individual. In fact, epigenetic studies show that people’s genetics can be predicted by their ancestors’ exposure to sustained poverty and social inequality [78]. In this case, claiming that “poverty is in genetics” is not saying that there is a genetic cause but rather that these particular genetics could also be the consequence or correlation of ancestral poverty. In the public debate around poverty, there is not always a clear distinction between causes, correlates, and consequences.

We believe that bringing the aspect of controllability into the discussion helps shift the focus from where the cause of poverty lies (within or outside the person) to who is responsible and who can take action (internal or external controllability). Leaving aside the discussions of internal–external locus seems reasonable in the explanation of a phenomenon that emerges from the interaction of person–situation or person–society and is thus an inherently social issue. Simultaneously, as the internal-or-external locus discussion sometimes leads to a mere description of reality, foregrounding debates on controllability and responsibility can be a first step to engaging in actions to actually reduce poverty.

### 4.3. From Poverty to Inequality

In the social sciences, the phenomenon of poverty has acquired multiple conceptualisations [79]. Some studies, for example, rely on the concept of poverty as a lack of the financial and material goods necessary to live in a certain society, while others adopt a multidimensional approach, defining poverty as an unjust deprivation of social, economic, and cultural rights (including housing, work, food security, clothing, education, and healthcare, among others) affecting the development of human capacities and social integration [76,77]. Not only in academia but also for research participants, poverty is far from an unequivocal concept. Research has shown that “poverty” and “the poor” employed as a general stimulus can bring to mind images and associations ranging from people suffering from hunger to homeless people, from people experiencing a lack of money to a lack of rights [80]. Thus, it is worth asking: attributions of what kind of poverty are we investigating?

In fact, a critique of mainstream research on poverty attributions is that studies have almost exclusively relied on a generic conceptualisation of poverty and “the poor” as a homogeneous group [39,64,81,82], failing to acknowledge that different definitions and types of poverty might trigger different causal interpretations [39]. In our review, when reading full texts, we also often found it difficult to identify attributions of what type of poverty were being captured since, in most cases, participants were asked to explain the causes of poverty without further specification (e.g., *Please rate the importance of each of those factors as causes of poverty from 1 (not at all important) to 5 (extremely important)* [26]). However, we assume that many studies implicitly deal with monetary poverty attributions, since when listing the causes of poverty in their measurements, they largely mention economic aspects (*“lack of thrift and proper money management”, “prejudice and discrimination in promotion and wages”* [26]).

In public opinion research, where opinions (in this case, attributions) can change easily depending on how survey questions are framed [83], we believe that the use of “poverty” as a prompt to explore explanations for economic outcomes may have downsides. These are not only due to its polysemic connotation as explained above, but also to its framing (as in the case of monetary poverty) of economic suffering. Accordingly, we instead see potential in focusing on the concept of *economic inequality* in the study of attributions, which may bring in a more process-oriented and relational understanding of individuals’ economic plight. While poverty tends to be understood as a descriptive concept of the state of lack experienced by a person or group, focusing on who suffers from poverty [80], inequality is by definition a relational concept with a focus on the dynamic between the disadvantaged and the advantaged. In that sense, since inequality always implies a comparison group, it does not allow the system of relationships from which poverty arises to remain invisible. By asking respondents to explain economic (pay, income, or wealth) differences, it is possible to explore attributions of economic suffering without neglecting the social background in which it occurs.

Our review identifies attributions for inequality as a major gap in this field of study. The results show a strikingly small number of studies in this regard and, in our review, no scale measuring attributions for economic inequality was found. Considering the arguments outlined above, the field would benefit from incorporating the concept of inequality in future research to be able to adequately address the societal challenges posed by it today (see [84] for a recent qualitative study on global inequality attributions).

### 4.4. Limitations

The main limitation of our review is that, due to the search criteria employed, we might not have covered the entire landscape of studies on attributions for poverty and economic inequality. Since we restricted the results to articles mentioning the keywords in the title, we might have missed out on publications which, for example, did not mention attributions explicitly in the title, but rather referred to attitudes or explanations. However, the current study still presents an extensive overview of the literature, and the trends we identified, namely a WEIRD bias in samples, the preponderance of certain theoretical approaches, and a lack of scales focusing on the majority world and the concept of inequality, are unlikely to be affected by the studies that were potentially omitted.

## 5. Conclusions

In Weiner’s article “Wither attribution theory?”, the author points out that the field needs to tap into new areas and expand to new phenomena the theory originally did not address [40]. He calls for the introduction of new theoretical challenges that force reconceptualisation and rethinking in the realm of attribution research. The current systematic review makes a substantial contribution to this by pointing to various gaps and limitations of the instruments employed to measure poverty and inequality attributions over the years. It also serves as a call for researchers to critically reflect on how we understand and study economic plights within countries and globally.

Our review analyses *what*, *how*, and *who* is being measured in the literature on poverty and inequality attribution. We found that the current scales focus on poverty rather than inequality and therefore, given that poverty is not always understood as a relational concept, run the risk of making the relationship between the rich and the poor as well as the dynamics leading to the perpetuation of economic plights invisible. Our results further show that most scales to date are based on the structural and individualistic dimensions introduced by Feagin [10] and suggest that the field could benefit from a stronger focus on controllability and responsibility as in the taxonomy by Weiner [22,23]. Finally, we identified that most scales were employed to measure domestic poverty, developed from a WEIRD perspective and applied to WEIRD populations [45]. There is a lack of consideration of poverty and inequality as a global issue, especially global inequality. This seems particularly relevant today—even though global poverty has decreased overall since the early 1990s [85], economic inequality is at a historical peak [86]. Furthermore, as Piketty [87] points out, inequalities have actually widened in times of global economic growth.

On a different note, the importance of controllability and responsibility as well as the concept of inequality as discussed here come with certain implications for policymakers. Focusing on those in control and considering both sides of the divide between the advantaged and the disadvantaged makes the systematic nature in which economic and in particular global inequality is maintained and reproduced clearly visible. To break the cycle in which this inequality is reproduced over and over again would mean shifting from charity to justice, as Hickel [88] puts it. Charity and aid cannot alleviate poverty since the financial resources for such assistance stem from the very processes leading to poverty in the first place [88]. Importantly, they also do not put the disadvantaged back in control of their situation but instead reproduce and stabilise existing hierarchies and dependencies. Hence, Hickel [88] calls for changing the rules that perpetuate poverty by implementing measures such as abolishing debt burdens, democratising global institutions, and introducing a universal basic income, amongst others. However, these truly empowering measures will likely only come onto the table once politicians as well as the broader public cease to attribute responsibility to the poor and poor countries themselves.

The current human development report by the United Nations Development Programme refers to a new uncertainty complex that we live in today, with humanity facing a variety of crises, some of them very much related to (global) inequality [1]. However, these uncertainties also constitute a chance to reimagine, renew, and adapt to today’s challenges. The research on inequality attributions holds the potential to be part of this path forward.

## Data Availability

Not applicable.

## Appendix A

**Table A1:** Searches in free and paid databases.

Search Engine and Databases	Keyword Combination	Results
Google Scholar	poverty AND attributions	113
	poverty AND attribution	40
	poor AND attributions	50
	poor AND attribution	44
	inequality AND attributions	23
	inequality AND attribution	16
	pobreza AND atribuciones	28
	pobreza AND atribución	3
	pobre AND atribuciones	1
	pobre AND atribución	0
	desigualdad AND atribuciones	1
	desigualdad AND atribución	1
	Total	320
EBSCO host (including APA PsycArticles, Psychology and Behavioral Sciences Collection, APA PsycInfo, PSYNDEX Literature with PSYNDEX Tests)	poverty AND attributions	73
	poverty AND attribution	73
	poor AND attributions	31
	poor AND attribution	31
	inequality AND attributions	14
	inequality AND attribution	14
	pobreza AND atribuciones	4
	pobreza AND atribución	0
	pobre AND atribuciones	1
	pobre AND atribución	0
	desigualdad AND atribuciones	0
	desigualdad AND atribución	0
	Total	241

**Table A2:** Studies measuring poverty attributions from 1972 to 2023.

№	Authors and Year of Publication	Scope of Attributions	Sample Country	Sample Size and Profile	Theoretical Reference	№ of Items and Scale Response Anchors	Dimensions and Their Reliability *
1	Abouchedid and Nasser (2002) [89]	Domestic poverty or without specification	Lebanon and Portugal	372 university students	Feagin (1972; 1975) [10,34]	15 items, 1–5 (disagree–agree)	Lebanon: structural (α = 0.63), individualist (α = 0.67), fatalist (α = 0.67); Portugal: structural (α = 0.54), individualist (α = 0.70), fatalist (α = 0.77)
2	Alcañiz-Colomer, Moya, and Valor-Segura (2023) [90]	Domestic poverty or without specification	Spain	484 (study 1, survey respondents); 256 (study 2, undergraduate students); 358 (study 3, survey respondents)	Furnham, (1982) [31], Weiner et al., (2011) [23]	20 items, 1–5 (strongly disagree–strongly agree) + 4 items for the Spanish context	Individualistic (α = 0.80/0.83/0.71), structural (α = 0.81/0.81)
3	Bai, Xu, Yang, and Guo (2023) [91]	Domestic poverty or without specification	China	448 (study 1)	Li (2014) [92]	16 items, 1–7 (totally disagree–totally agree)	Internal (α = 0.79), external (α = 0.76)
4	Bennett, Raiz, and Davis (2016) [27]	Domestic poverty or without specification	USA	209 social workers	Bullock (2004) [68], Bullock et al., (2003a) [29], Weiss-Gal and Gal (2007) [93]	33 items, 1–6 (fully agree–fully disagree)	Individual (α = 0.94), structural (α = 0.88), cultural (α = 0.77)
5	Bergmann and Todd (2019) [94]	Domestic poverty or without specification	USA	189 (study 1) and 646 (study 2) university students	Cozzarelli et al., (2001) [26]	13 items, 1–5 (not important at all–extremely important)	Internal (α = 0.83), external (α = 0.80)
6	Bobbio, Canova, and Manganelli (2010) [95]	Domestic poverty or without specification	Italy	181 university students	Feagin (1972) [10], Smith and Stone (1989) [37]	12 items, 1–5 (not important at all–extremely important)	Internal/individualistic (α = 0.82), external/structuralistic (α = 0.74)
7	Bradley and Cole (2002) [96]	Domestic poverty or without specification	Canada and the USA	714 survey respondents aged 18 or older	Feagin (1975) [34]	11 items, 1–3 (very important–not important at all)	Internal (α = 0.60), external (α = 0.62)
8	Bullock (1999) [30]	Domestic poverty or without specification	USA	236 survey respondents	Furnham (1982) [31]	16 items, 1–7 (strongly disagree–strongly agree)	Individualistic, structural, structural–fatalistic
9	Bullock, Williams, and Limbert (2003) [29]	Domestic poverty or without specification	USA	131 university students	Bullock (1999) [30], Cozzarelli et al., (2001) [26], Furnham (1982) [31]	45 items, 1–7 (disagree–agree)	Structural (α = 0.91), individualistic/poverty culture (α = 0.91), fatalistic/structural (α = 0.72)
10	Canto, Perles, and San Martín (2012) [97]	Global poverty	Spain	300 university students	Hine and Montiel (1999) [7], adapted by Betancor et al., (2002) [98]	22 items, 1–6 (fully disagree–fully agree)	Structural, personal, fatalistic
11	Carr, Taef, De M.S. Ribeiro and MacLachlan (1998) [99]	Global poverty	Australia and Brazil	100 textile workers	Harper et al., (1990) [32]	16 items, 1–5 (disagree–agree)	Nature, the poor, local governments, exploitation
12	Carr and MacLachlan (1998) [100]	Global poverty	Australia and Malawi	582 university students	Harper et al., (1990) [32]	20 items, 1–5 (disagree–agree)	Blame the poor, blame international exploitation, blame nature, blame third-world governments
13	Castillo and Rivera-Gutiérrez (2018) [74]	Domestic poverty or without specification	Chile	1245 survey respondents aged 18 or older	Feagin (1972) [10]	5 items, 1–5 (never–always)	Internal/individual, external/sociocultural
14	Cheng and Ngok (2023) [101]	Domestic poverty or without specification	China	10,855/10,678 survey respondents	Feagin (1972) [10]	5 items (dummy variables)	Individualistic, structural, fatalistic
15	Cojanu and Stroe (2017) [102]	Domestic poverty or without specification	Romania	600 beneficiaries of guaranteed minimum income	N/A	10 items, 1–2 (ordinal scale)	Individual, structural/societal, fatalistic
16	Cozzarelli, Wilkinson, and Tagler (2001) [26]	Domestic poverty or without specification	USA	209 university students	Feagin (1972) [10], Smith and Stone (1989) [37]	22 items, 1–5 (not important at all–very important)	Internal (α = 0.75), external (α = 0.79), cultural (α = 0.65)
17	da Costa and Dias (2013) [57]	Domestic poverty or without specification	13 European countries	15,504 respondents above age 15	N/A	11 items (dummy variables)	Individualistic/internal, structural, Fatalist
18	Davidai (2018) [103]	Domestic poverty or without specification	USA	397 survey respondents	Kluegel and Smith (1986) [36]	7 items, 1–7 (not so important–extremely important)	Internal, external
19	Engler, Strassle, and Steck (2019) [104]	Domestic poverty or without specification	USA	161 primary and secondary education administrative employees	Cozzarelli et al., (2001) [26]	22 items	Internal, external
20	Frei, Castillo, Herrera, and Suárez (2020) [105]	Domestic poverty or without specification	Chile	2954 survey respondents aged 18 or older	N/A	10 items, 1–2 (ordinal scale)	Internal, external, ambivalent
21	Gatica and Navarro-Lashayas (2019) [106]	Domestic poverty or without specification	Spain	184 university students	Bullock et al., (2003) [29]	32 items, 1–5 (not important–very important)	Sociostructural (α = 0.91), individual (α = 0.91), fatalistic (α = 0.72)
22	Generalao (2005) [107]	Domestic poverty or without specification	Philippines	373 housewives (145 from rural areas and 228 from urban areas)	Weiner (1985) [14]	7 items, 1–5 (semantic differential scale)	Locus, controllability, Stability
23	Gonzalez, Macchia, and Whillans (2022) [108]	Domestic inequality or without specification	USA	200 survey respondents	Hussak and Cimpian (2015) [109]	3 items (forced choice)	Uncontrollable dispositional/controllable dispositional/uncontrollable situational
24	Griffin and Oheneba-Sakyi (1993) [110]	Domestic poverty or without specification	USA	207 undergraduate students	N/A	1 item (dummy variable)	Individual
25	Habibov, Cheung, Auchynnikava, and Fan (2017) [58]	Domestic poverty or without specification	24 European and Asian countries	37,307 survey respondents aged 17 or older	N/A	1 item (dummy variable)	Structural
26	Halik, Malek, Bahari, Matshah, and Webley (2012) [111]	Domestic poverty or without specification	Malaysia	124 university students	Feagin (1972) [10], Furnham (1982) [31]	16 items, 1–5 (strongly disagree–strongly agree)	Individualistic (α = 0.71), structural (α = 0.63), fatalistic (α = 0.58)
27	Heaven (1989) [112]	Domestic poverty or without specification	Australia	285 survey respondents aged 18 or older	Feagin (1972) [10], Furnham (1982) [31]	11 items	Societal (α = 0.72), negative individualistic (α = 0.75), characterological (α = 0.66)
28	Hill, Toft, Garrett, Ferguson, and Kuechler (2016) [113]	Domestic poverty or without specification	USA	337 university students	Feagin (1972; 1975) [10,34]	7 items, 1–5 (agree–disagree)	Individual (α = 0.66), structural (α = 0.70)
29	Husz, Kopasz, and Medgyesi (2022) [114]	Domestic poverty or without specification	Hungary	600 social workers	Feagin (1972) [10]	10 items, 1–4 (strongly agree–strongly disagree)	Structural, individualistic
30	Ige and Nekhwevha (2012) [115]	Domestic poverty or without specification	South Africa	383 survey respondents	Feagin (1972) [10]	38 items, 1–5 (strongly disagree–strongly agree)	Structural (α = 0.86), individual (α = 0.91), fatalistic (α = 0.85)
31	Kafetsios and Kateri (2022) [116]	Domestic inequality or without specification	Greece	846 survey respondents	N/A	12 items	Dispositional, contextual
32	Kitchens, Ricks, and Hannor-Walker (2020) [117]	Domestic poverty or without specification	USA	91 university students	Bullock (1999) [30]	36 items, 1–5 (not at all important as a cause of poverty–extremely important as a cause of poverty)	Individualistic (α = 0.91), structuralistic (α = 0.91), fatalistic (α = 0.72)
33	Landmane and Reņģe (2010) [118]	Domestic poverty or without specification	Latvia	202 women	Bullock et al., (2003) [29], Bullock (2004) [68]	30 items, 1–5 (strong disagreement–strong agreement)	Family/fatalistic (α = 0.86), individualistic (α = 0.79), structural (α = 0.77)
34	Lee, Park, Rhee, Kim, Lee, Ha, Baik, and Ahn (2021) [119]	Domestic poverty or without specification	South Korea, Japan, USA	2213 survey respondents representative of each country’s population	Feagin (1972, 1975) [10,34]	8 items, 1–5 (strongly disagree–strongly agree)	Individualistic (α = 0.79), societal (α = 0.64)
35	Ljubotina and Ljubotina (2007) [120]	Domestic poverty or without specification	Croatia	365 university students	Feagin (1975) [34]	24 items, 1–5 (completely disagree–completely agree)	Individual (α = 0.76), structural (α = 0.79), fatalistic (α = 0.70), micro-environmental/cultural (α = 0.65)
36	McWha and Carr (2009) [121]	Global poverty	New Zealand	171 university students	Harper et al., (1990) [32]	17 items, 1–5 (strongly disagree–strongly agree)	Blame the poor (α = 0.77), blame third-world governments (α = 0.70), blame nature (α = 0.56)
37	Mickelson and Hazlett (2014) [54]	Domestic poverty or without specification	USA	66 low-income women with at least one child aged 1–6 years	Bullock et al., (2003) [29]	37 items, 1–5 (did not contribute at all–contributed a lot)	Structural (α = 0.90), individualistic (α = 0.76), children (α = 0.71), romantic relationships (α = 0.66), fatalistic (α = 0.65)
38	Murry, Brody, Brown, Wisenbaker, Cutrona, and Simons (2002) [122]	Domestic poverty or without specification	USA	96 single mothers who receive government welfare	Conger (1995) [123]	16 items, 1–4 (agree–disagree)	External (α total = 0.76)
39	Nasser (2007) [124]	Domestic poverty or without specification	Lebanon	242 high-school students	Feagin (1972) [10]	N/A	Structuralistic, individualistic, fatalistic
40	Nasser and Abouchedid (2001) [125]	Domestic poverty or without specification	Lebanon	232 university students	Feagin (1972) [10], Hunt (1996) [65], Morcol (1997) [126], Griffin and Oheneba-Sakyi (1993) [110], Williamson (1974) [127]	15 items, 1–5 (strongly agree–strongly disagree)	Structuralist (α = 0.70), individualist status quo (α = 0.60), fatalist (α = 0.70), individual blaming the poor/societal (α = 0.50)
41	Nasser and Abouchedid (2006) [128]	Domestic poverty or without specification	Lebanon, South Africa	443 university students	Feagin (1972) [10]	15 items, 1–5 (strongly agree–strongly disagree)	Individualism (α = 0.71), fatalism (α = 0.62), structuralism (α = 0.50)
42	Nasser, Singhal, and Abouchedid (2005) [129]	Domestic poverty or without specification	India	365 high-school and university students	Feagin (1972) [10], adapted by Nasser and Abouchedid (2001) [125]	17 items, 1–5 (fully agree–fully disagree)	Individualistic, structural, fatalistic (α total = 0.63)
43	Nelson and Joselus (2023) [130]	Domestic inequality or without specification	USA	448 survey respondents	Peffley, Hurwitz, and Mondak (2017) [131]	7 items, 1–6 (not important at all–extremely important)	Internal (α = 0.915), cultural (α = 0.906), external
44	Niemelä (2011) [39]	Domestic poverty or without specification	Finland	2006 social security officials and other citizens	Feagin (1972) [10], van Oorschot and Halman (2000) [50], Saunders (2003) [132]	11 items, 1–5 (strongly agree–strongly disagree)	Individual, individual–structural, structural, fatalistic
45	Norcia, Castellani, and Rissotto (2010) [133]	Domestic poverty or without specification	Italy	1914 survey respondents	N/A	7 items, 1–5 (never–very often)	Internal, external, fatalism
46	Norcia and Risotto (2011) [134]	Domestic poverty or without specification	Italy	1914 survey respondents	N/A	7 items, 1–5	Powerful others, chance, internal
47	Norcia and Rissotto (2015) [55]	Domestic poverty or without specification	Italy	992 survey respondents	N/A	8 items, 1–5	Internal (α = 0.57), powerful other (α = 0.66), chance (α = 0.63)
48	Osborne and Weiner (2015) [22]	Domestic poverty or without specification	New Zealand and the USA	315 university students	McAuley et al., (1992) [25]	12 items, 1–7 (semantic differential scale)	Locus (α = 0.79), stability (α = 0.65), personal control (α = 0.83), other control (α = 0.71)
49	Özpinar and Akdede (2022) [135]	Domestic poverty or without specification	Turkey	1110 participants	Feagin (1972) [10]	7 items (dummy variables)	Individualistic, Structural, Fatalistic
50	Pandey, Sinha, Prakash, and Tripathi (1982) [136]	Domestic poverty or without specification	India	90 university students	Sinha et al., (1980) [137]	8 items, 1–5 (completely disagree–completely agree)	Self, fate, governmental policies, economic dominance
51	Piff, Wiwad, Robinson, Aknin, Mercier, and Shariff (2020) [8]	Domestic poverty or without specification	USA	602 survey respondents	Feagin (1972) [10], Kluegel and Smith (1986) [36]	12 items, 1–5 (not so important–extremely important)	Situational attributions (α = 0.85), dispositional attributions (α = 0.79)
52	Reutter, Veenstra, Stewart, Raphael, Love, Makwarimba, and McMurray (2006) [138]	Domestic poverty or without specification	Canada	1671 survey respondents	van Oorschot and Halman (2000) [50]	5 items, 1–5 (strongly disagree–strongly agree)	Structural, sociocultural, individualistic, fatalistic
53	Reyna, Acosta, Saavedra, and Correa (2018) [139]	Domestic poverty or without specification	Argentina	280 survey respondents	Cozzarelli et al., (2001) [26]	23 items, 1–5 (not important for poverty–extremely important as a poverty cause)	Internal (α = 0.77), sociostructural (α = 0.76), fatalistic (α = 0.69)
54	Reyna and Reparaz (2014) [28]	Domestic poverty or without specification	Argentina	177 university students	Cozzarelli et al., (2001) [26]	23 items, 1–5 (not important for poverty–extremely important as a poverty cause)	Internal (α = 0.74), external (α = 0.73), cultural (α = 0.79)
55	Ríos-Rodríguez, Moreno-Jiménez, and Vallejo Martín (2022) [140]	Global poverty	Spain	720 survey respondents	N/A	17 items	Cultural learning (α = 0.80), factic, (α = 0.82), deterministic (α = 0.71)
56	Robinson (2011) [53]	Domestic poverty or without specification	USA	839 survey respondents (431 social workers and 408 school teachers)	Feagin (1975) [34], Kluegel and Smith (1982) [36]	11 items	Individual (α = 0.70), structural (α = 0.72), psycho/medical (α = 0.63), family/morals (α = 0.68)
57	Sainz, García-Castro, Jiménez-Moya, and Lobato (2023) [141]	Domestic poverty or without specification	Mexico	523 survey respondents	Cozzarelli et al., (2001) [26]	11 items, 1–7 (not at all–completely)	Internal (α = 0.88), external (α = 0.80)
58	Schneider and Castillo (2015) [48]	Domestic poverty or without specification	Germany	3059 survey respondents (715 from East Germany and 2344 from West Germany)	N/A	5 items, 1–5 (very often–never)	Internal, external
59	Segretin, Reyna, and Lipina (2022) [142]	Domestic poverty or without specification	Argentina	1659 survey respondents	Bolitho et al., (2007) [143], Cozzarelli et al., (2001) [26], Ige and Nekhwevha, (2014) [144], Reyna and Reparaz, (2014) [28], Vázquez et al., (2010) [33], Weiss-Gal et al., (2009) [52]	32 items, 1–5 (not important for poverty–extremely important as a poverty cause)	Internal or individualistic (α = 0.90), external or structural (α = 0.90)
60	Sigelman (2012) [56]	Domestic poverty or without specification	USA	88 primary education students	N/A	9 items, 1–5 (no–yes)	Competence (α = 0.79), social attractiveness (α = 0.81), physical attractiveness (α = 0.68)
61	Smith and Kluegel (1979) [49]	Domestic poverty or without specification	USA	175 respondents aged 18 or older	Feagin (1972) [10]	N/A	Structural (α = 0.62), individual (α = 0.77)
62	Stoeffler, Kauffman, and Joseph (2021) [145]	Domestic poverty or without specification	USA	1037 social work educators	Bullock et al., (2003) [29]	41 items, 1–7 (strong agreement–strong disagreement)	Structural, individual, fatalistic
63	Swami, Voracek, Furnham, Robinson and Tran (2023) [146]	Domestic poverty or without specification	UK	392 respondents	Yun and Weaver (2010) [147]	21 items, 1–5 (fully disagree–fully agree)	Individualistic; discriminatory; structural (reliability across subscales, McDonald’s ω = 0.91)
64	Terol-Cantero, Martin-Aragón Gelabert, Costa-López, Manchón López, and Vázquez-Rodríguez (2023) [148]	Domestic poverty or without specification	Spain	278 university students	Cozzarelli et al., (2001) [26], Reyna and Reparaz (2014) [28]	23 items, 1–5 (not at all important–extremely important)	Cultural (α = 0.69), internal (α = 0.73), external (α = 0.77)
65	Toporek and Pope-Davis (2005) [66]	Domestic poverty or without specification	USA	158 master’s students	Smith and Stone (1989) [37]	19 items, 1–3 (is not important–is very important)	Individualism (α = 77), structuralism/situationalism (α = 72).
66	Vázquez and Panadero (2007) [149]	Global poverty	Spain and Nicaragua	294 university students	Harper et al., (1990) [32]	25 items, 1–5 (fully disagree–fully agree)	Dispositional, situational
67	Vázquez and Panadero (2009) [150]	Global poverty	Spain and Nicaragua	294 university students	Harper et al., (1990) [32]	25 items, 1–5 (fully disagree–fully agree)	Dispositional, situational
68	Vázquez, Panadero, Pascual, and Ordoñez (2017) [59]	Global poverty	Spain	1092 university students	Harper (2002) [32], Hine et al., (2005) [151], Vázquez and Panadero (2009) [150]	50 items, –2–+2 (fully disagree–fully agree)	Fault of the world economic structure; fault of fate, nature, cultural habits, and political misconduct; fault of the developing countries’ population
69	Vilchis Carrillo (2022) [152]	Domestic poverty or without specification	Mexico	1403 survey respondents	N/A	3 items (dichotomous response)	Structural, individualistic, fatalistic
70	Waddell, Wright, Mendel, Dys-Steenbergen, and Bahrami (2023) [9]	Domestic poverty or without specification	Canada	337 undergraduate students (study 1), 203 undergraduate students (study 2)	Furnham (1982) [31]	9/10 items, 1–7 (strongly disagree–strongly agree)	Internal (α = 0.77), external (α = 0.65)
71	Weiss-Gal, Gal, Benyamini, Ginzburg, Savaya, and Peled (2009) [52]	Domestic poverty or without specification	Israel	811 survey respondents (401 clients and 410 social workers)	Weiss-Gal (2005) [51], Weiss-Gal et al., (2003) [153], Bullock et al., (2003) [29]	25 items, 1–5 (strongly disagree–strongly agree)	Psychological (α = 0.89), motivational (α = 0.87), sociostructural (α = 0.82), fatalistic (α = 0.78)
72	Wollie (2009) [154]	Domestic poverty or without specification	Ethiopia	460 high-school and university students	Feagin (1972) [10], Nasser and Abouchedid (2001) [125], Nasser et al., (2005) [129]	39 items, 1–5 (strongly disagree–strongly agree)	Individualistic, structural, fatalistic
73	Yeboah and Ernest (2012) [155]	Domestic poverty or without specification	Ghana	147 university students	N/A	N/A	Individual, structural, fatalistic
74	Yúdica, Bastias, and Etchezahar (2021) [44]	Domestic poverty or without specification	Argentina	331 secondary school students	Gatica et al., (2017) [156], based on Bullock et al., (2003) [29]	32 items; 1–5 (not at all important–very important)	Individualistic (α = 0.81); structural (α = 0.80); fatalistic (α = 0.61)

Note. * Reliabilities were not available for all scales.
